# Adult granulosa cell tumours of the testis analogous to ovarian counterparts are exceptionally rare: analysis of a multicentric series and review of the literature

**DOI:** 10.1111/his.70048

**Published:** 2025-11-20

**Authors:** Costantino Ricci, Dario de Biase, Thais Maloberti, Agnese Orsatti, Thomas M Ulbright, Muhammad T Idrees, Esther Oliva, Kristine Cornejo, João Lobo, Kvetoslava Michalova, Maria Rosaria Raspollini, Sean R Williamson, Geert JLH van Leenders, Chia‐Sui Kao, Fiona Maclean, Ankur R Sangoi, Adeboye O Osunkoya, Michelangelo Fiorentino, Antonio De Leo, Giovanni Tallini, Andres Martin Acosta

**Affiliations:** ^1^ Pathology Unit, DIAP‐Dipartimento Interaziendale di Anatomia Patologica di Bologna Maggiore Hospital‐AUSL Bologna Bologna Italy; ^2^ Solid Tumor Molecular Pathology Laboratory IRCCS Azienda Ospedaliero‐Universitaria di Bologna Bologna Italy; ^3^ Department of Pharmacy and Biotechnology (FaBiT) University of Bologna Bologna Italy; ^4^ Department of Pathology and Laboratory Medicine Indiana University School of Medicine Indianapolis Indiana USA; ^5^ Department of Pathology Massachusetts General Hospital Boston Massachusetts USA; ^6^ Department of Pathology Portuguese Oncology Institute of Porto (IPOP) Porto Portugal; ^7^ Cancer Biology and Epigenetics Group, Research Center of IPO Porto (GEBC CI‐IPOP) Portuguese Oncology Institute of Porto (IPO Porto)/Porto Comprehensive Cancer Center (P.CCC) Porto Portugal; ^8^ Department of Pathology and Molecular Immunology, ICBAS‐School of Medicine and Biomedical Sciences University of Porto (ICBAS‐UP) Porto Portugal; ^9^ Department of Pathology Charles University, Faculty of Medicine in Plzeň, Bioptical Laboratory, Ltd Plzeň Czech Republic; ^10^ Department of Histopathology and Molecular Diagnostics Careggi University Hospital Florence Italy; ^11^ Pathology and Laboratory Medicine Institute The Cleveland Clinic Cleveland Ohio USA; ^12^ Department of Pathology, Erasmus Medical Center Erasmus University Rotterdam Netherlands; ^13^ Douglass Hanly Moir Pathology Macquarie Park New South Wales Australia; ^14^ Department of Pathology Stanford University Stanford California USA; ^15^ Department of Pathology and Urology Emory University School of Medicine Atlanta Georgia USA; ^16^ Department of Medical and Surgical Sciences (DIMEC) University of Bologna Bologna Italy

**Keywords:** *CTNNB1*, *CTNNB1* mutation, *FOXL2*, *FOXL2* mutation, NGS, sex cord‐stromal tumours, testicular adult granulosa cell tumour

## Abstract

**Aims:**

Testicular adult granulosa cell tumours (AGCTs) are rare and show several clinical–pathological differences with their ovarian counterparts. In a limited number of prior studies, *FOXL2* p.Cys134Trp, the hallmark molecular alteration of ovarian AGCT, appeared to be infrequent in testicular AGCTs. However, the number of cases analysed to date is relatively small.

**Methods and results:**

Twenty testicular AGCTs were analysed *de novo* using two different next‐generation sequencing (NGS) panels that cover sex cord‐stromal tumour (SCST)–relevant genes, including *FOXL2* , *CTNNB1* , *FH* and *DICER1* . Among 12 tumours (12/20; 60%) that were sequenced successfully, none harboured *FOXL2* mutations. Eight tumours (8/12, 66.7%) showed a wild‐type (WT) status for all genes assessed with the panels. Three tumours harboured pathogenic or likely pathogenic *CTNNB1* alterations. One of these exhibited predominant spindle cell morphology, while the other two showed focal tubular architecture. Immunohistochemistry performed in one of these tumours with available material showed β‐catenin expression in ~70% of tumor cell nuclei. The remaining AGCTs showed variants of uncertain significance (likely benign) in *KIT* and *MED12* . Considering the tumors asseseed in this study and those previously reported in the literature, only 2 of 29 neoplasms classified as testicular AGCTs have shown a *FOXL2* p.Cys134Trp mutation to date.

**Conclusions:**

The present study confirms that SCSTs classified as AGCTs differ from their ovarian counterparts in that they largely lack *FOXL2* mutations.

## Graphical abstract

Assessment of twenty testicular AGCTs with two different next‐generation sequencing (NGS) panels reveals differences with ovarian AGCTs, including absence of hotspot *FOXL2* variants.

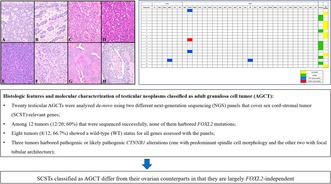

**Abbreviations**
AGCT/AGCTs: adult granulosa cell tumour/tumours
CDS: entire coding sequence
FFPE: formalin‐fixed paraffin‐embedded
FISH: fluorescence *in situ* hybridisation
GOF: gain‐of‐function
H&E: haematoxylin and eosin
SCST/SCSTs: sex cord‐stromal tumour/tumours
VAF: variant allele frequency

## Introduction

Testicular adult granulosa cell tumours (AGCTs) are very rare and represent 0.5% of all sex cord‐stromal tumours (SCSTs) in male patients.^1^, ^2^, ^3^ In contrast, ovarian AGCT is the most common SCST in adult female patients, accounting for up to 2%–5% of all ovarian neoplasms.^4^, ^5^, ^6^ In male patients, AGCT typically presents as a unilateral, slowly growing, painless mass but some may experience manifestations of sex hormone overproduction such as erectile dysfunction, gynecomastia and decreased libido.^1^, ^2^, ^3^ This represents another significant difference with ovarian homonyms, which are almost invariably characterised by sex hormone overproduction leading to metrorrhagia, postmenopausal uterine/vaginal bleeding, endometrial hyperplasia, endometrial endometrioid carcinoma, virilisation and hirsutism.^4^, ^5^, ^6^ Approximately 10%–20% of testicular AGCT show malignant clinical behaviour, metastasizing to retroperitoneal lymph nodes, liver, lung and bone.^1^, ^2^, ^3^ In comparison, a relatively higher proportion of ovarian AGCTs is malignant (20%–30%), typically developing late metastases to the peritoneum, omentum, liver and lung.^4^, ^5^, ^6^ The clinical and, to some degree, pathological differences between ovarian and testicular AGCTs likely reflect differences in underlying pathogenic mechanisms.^7^, ^8^, ^9^, ^10^, ^11^, ^12^, ^13^, ^14^ The genomic features of ovarian AGCTs have been extensively studied, establishing *FOXL2* as the central oncogenic driver of the disease.^7^, ^8^, ^9^ Specifically, the overwhelming majority of ovarian AGCTs (93–97%) harbour a hotspot gain‐of‐function (GOF) *FOXL2* variant [c.7558C > T (p.Cys134Trp)].^7^, ^8^, ^9^ Significantly less is known about the genomic alterations of testicular AGCTs, which have only been interrogated in individual case studies or small series previously.^10^, ^11^, ^12^, ^13^, ^14^, ^15^ These data suggest that testicular AGCTs are markedly different from ovarian counterparts in that *FOXL2* alterations appear to be rare, with no highly recurrent molecular alterations being identified to date.^7^, ^8^, ^9^, ^10^, ^11^, ^12^, ^13^, ^14^, ^15^, ^16^, ^17^ In this study, we interrogated a multi‐institutional series of testicular AGCTs to further characterise their mutational landscape.

## Materials and methods

### Identification of cases, review of slides and clinical–pathological data

The pathology databases of nine institutions (Indiana University School of Medicine, Massachusetts General Hospital, Portuguese Oncology Institute of Porto, Faculty of Medicine in Plzeň, Careggi University Hospital in Florence, Cleveland Clinic, Emory University Hospital, Erasmus Medical Center in Rotterdam, Douglass Hanly Moir Pathology in Macquarie Park) and personal consultation files of one of the authors (TMU) were queried for reports of testicular SCSTs compatible with AGCTs (i.e., definitive or possible). Clinical data were retrieved from the corresponding electronic medical records and pathology reports. Selected slides of each case were reviewed at Indiana University (T.M.U. and A.M.A.). Only cases with available archival formalin‐fixed paraffin‐embedded (FFPE) material were included in the study. The following information (Table 1) was collected for each case: age at diagnosis, tumour size, laterality, clinical presentation, personal oncologic history and results of relevant immunohistochemical stains performed as part of the initial diagnostic workup (when available). The study was performed with the approval of the Institutional Review Board (IRB) of Indiana University (Protocol #18697, 2023).

**Table 1:** Clinical, pathological and immunohistochemical features of testicular adult granulosa cell tumors

Case number	Age at diagnosis (years)	Laterality	Clinical presentation	Personal oncologic history (including familial/genetic syndromes)	Diameter (mm)	Relevant IHC performed at the time of diagnosis	Other relevant data
1	65	Left	Testicular mass	No	26	SF‐1: + (diffuse); inhibin: −	No recurrence and/or metastasis at follow‐up (48 months)
2	59	Left	Testicular mass detected at physical exam performed during routine follow‐up for oncologic history	Previous rectal carcinoma and small bowel carcinoid; no familial/genetic syndromes	Not known	vimentin, inhibin A: +; PR: + (diffuse and strong in spindle‐cell component); ER: + (patchy and weak in spindle‐cell component); WT1: + (weak nuclear); CD99: + (cytoplasmic); CD56: + (focal); chromogranin A, synaptophysin, CK‐PAN, AE1/AE3, CK8/18, CK19, EMA, LCA, GFAP, CD34, S100, HMB45: −	No
3	45	Not known	Not known	Not known	95	Not performed/Not reported	Clinically malignant (the patient developed metastases)
4	33	Right	Small testicular mass on palpation	Not known	Not known	Calretinin and FOXL2: +++; inhibin, CK‐PAN, S100, vimentin: + (focal and weak), OCT4, SMA: −	No recurrence and/or metastasis at follow‐up (54 months)
5	37	Not known	Hydrocele	Not known	Not known	SF‐1: + (diffuse); inhibin, AE1/AE3: + (focal); CAM5.2: + (scattered cells); calretinin: −	No
6	22	Left	Testicular mass	No	Not known	Inhibin, calretinin, S100: + (focal); AE1/AE3: + (scattered cells); EMA, S100: −	No
7	70	Left	Testicular mass	No	Not known	Inhibin, AE1/AE3, calretinin: +; OCT4, SALL4: −	No
8	22	Not known	Testicular mass	Not known	Not known	Inhibin: +; calretinin, S100, SMA, AE1/AE3: −	No
9	27	Left	Hydrocele	No	Not known	Inhibin: +; calretinin: + (scattered cells); S100: + (focal areas); AE1/3, OCT4, synaptophysin: −	No
10	24	Left	Not known	Not known	Not known	Not performed/Not reported	Worrisome histopathologic features suggestive of malignancy
11	45	Left	Not known	Not known	110	Inhibin, SMA, SF‐1, FOXL2: +; calretinin: + (nests of cells); CD34, S100, SOX9: −	Worrisome histopathologic features suggestive of malignancy
12	38	Left	Not known	Not known	17	CK: + (dot‐like paranuclear pattern); S100, CD56: +	No worrisome histopathologic features suggestive of malignancy; originally diagnosed as unclassified SCST (consultation case diagnosed as AGCT by T.M.U)
13	35	Right	Ultrasound exam: testicular mass; normal serum marker levels	Not known	8	SF‐1, S100: + (diffuse); inhibin: + (weak and patchy); AE1/AE3, CK7, p63, GATA3, SMA, ‘germ cell markers (not specified)’: −; β‐catenin: − (only membrane)	No
14	43	Right	Microscopic hematuria; clinical exam: large and firm right testicle; CT scan: retroperitoneal lymphadenopathy	No (mother: breast cancer)	50	SF‐1, inhibin, SMA, calretinin: +; OCT4, CD45, SALL4, EMA: −; β‐catenin: − (only membrane); AE1/AE3: − (focal and weak)	No
15	65	Right	Painless testicular mass	History of marginal zone lymphoma	50	SF‐1, inhibin, ER, calretinin, AE1/AE3, vimentin, SMA: +; OCT4, SALL4: −; Ki‐67: <20%	No
16	54	Right	Not known	Not known	30	Not performed/Not reported	No worrisome histopathologic features suggestive of malignancy
17	49	Left	Gynecomastia and mastodynia − > ultrasound exam of the breast: ductal hyperplasia − > detection of testis nodule	No	Not known	SF‐1, inhibin, WT1: + (diffuse); Melan A, calretinin, CK‐PAN: + (multifocal); β‐catenin: − (only membrane); SALL4, OCT4, D2‐40, synaptophysin, chromogranin, SMA, S100: −; Ki‐67: 20%–30%	No recurrence and/or metastasis at follow‐up
18	71	Left	Testicular mass and one inguinal lymph node (suspicious for metastasis) at palpation	No	20	Not performed/Not reported	At histologic exam, involvement of rete testis but no extension outside testis, and clinically suspicious inguinal lymph node negative for metastasis
19	44	Right	Testicular mass	No	15	Calretinin, inhibin, vimentin, CD56, CD99, S100: +	No recurrence and/or metastasis at follow‐up
20	29	Not known	Not known	Not known	37	Inhibin, WT1, CD99: +; SMA: + (spindle‐cell component); OCT4: −	6 mitoses/10 HPF

Abbreviations: IHC, immunohistochemistry; NGS, next‐generation sequencing.

### NGS analysis

The samples were sequenced using two laboratory‐developed multi‐gene NGS panels following standard laboratory protocols at the Solid Tumour Molecular Pathology Laboratory, IRCCS Azienda Ospedaliero‐Universitaria di Bologna.^18^, ^19^ The first panel (panel‐1) interrogates 280 amplicons (29.24 kb, human reference sequence hg19/GRCh37) corresponding to the following genes: *FH* (NM_000143.4) (entire coding sequence – CDS), *FOXL2* (NM_023067.4) (CDS), *HMGA1* (CDS), *MED12* (CDS) (NM_005120.3), *TSC1* (NM_000368.5) (CDS) and *TSC2* (NM_000548.5) (CDS).^18^ The second panel (panel‐2) interrogates 330 amplicons (21.77 kb, human reference sequence hg19/GRCh37) of the following genes: *BRAF* (NM_004333.6) (Exons 11, 15), *CTNNB1* (NM_001904.4) (Exons 3, 7, 8), *DICER1* (NM_030621.4) (Exons 10, 21, 26, 27, 29), *DPYD* (NM_000110.4) (DPYD*2A, DPYD*13, *DPYD D949V*, HapB3), *EGFR* (NM_005228.5) (Exons 18, 19, 20, 21), *EIF1AX* (NM_001412.4) (Exons 1, 2), *GNA11* (NM_002067.5) (Exons 4, 5), *GNAQ* (NM_002072.5) (Exons 4, 5), *GNAS* (NM_000516.7) (Exons 8, 9), *H3F3A* (NM_001379043.1) (Exon 1), *HRAS* (NM_001130442.2) (Exons 2, 3, 4), *IDH1* (NM_005896.4) (Exon 4), *IDH2* (NM_002168.4) (Exon 4), *KIT* (NM_000222.3) (Exons 8, 9, 11, 13, 17), *KRAS* (NM_033360.4) (Exons 2, 3, 4), *MET* (NM_001127500.3) (Exons 2, 14), *NRAS* (NM_002524.5) (Exons 2, 3, 4), *PDGFRa* (NM_006206.6) (Exons 12, 14, 18), *PIK3CA* (NM_006218.4) (Exons 8, 10, 21), *PTEN* (NM_000314.8) (Exon 5), *RET* (NM_020975.6) (Exons 5, 8, 10, 11, 13, 14, 15, 16), *RNF43* (NM_017763.6) (Exons 2–9), *SMAD4* (NM_005359.6) (Exons 2–12), *TERT* (NM_198253.3) (promoter region, g.1295141–1295471), *TP53* (NM_000546.6) (Exons 2–11), *TSHR* (NM_000369.5) (Exons 1–10) and *VHL* (NM_000551.4) (Exons 1–3).^19^ DNA was extracted from FFPE tumour tissue dissected manually from glass slides using marked haematoxylin and eosin‐stained (H&E) sections as a reference. An input of 30 ng of DNA was used for library preparation with the AmpliSeq Plus Library Kit 2.0 (Thermo Fisher Scientific, Waltham, MA, USA).^19^ Templates were sequenced on an Ion 530 chip, and the results were analysed using the Ion Reporter tools (version 5.20, Thermo Fisher Scientific) and Genome Browser Tool (https://www.goldenhelix.com/).^19^ Only single‐nucleotide variants/Indels present in at least 10% of the analysed reads and observed in both strands were considered for variant calling.^19^ NGS results were assessed for clinical and biological relevance by two of the authors with expertise in molecular pathology (D.d.B., T.M.); the Varsome tool (https://varsome.com/, last access on April 2025) and Franklyn by Genoox (https://franklin.genoox.com/, last access on April 2025) were used to evaluate the American College of Medical Genetics and Genomics (ACMG) classification of each reported variant.^20^

## Results

### Case series: clinical–pathological features

**Figure 1:**
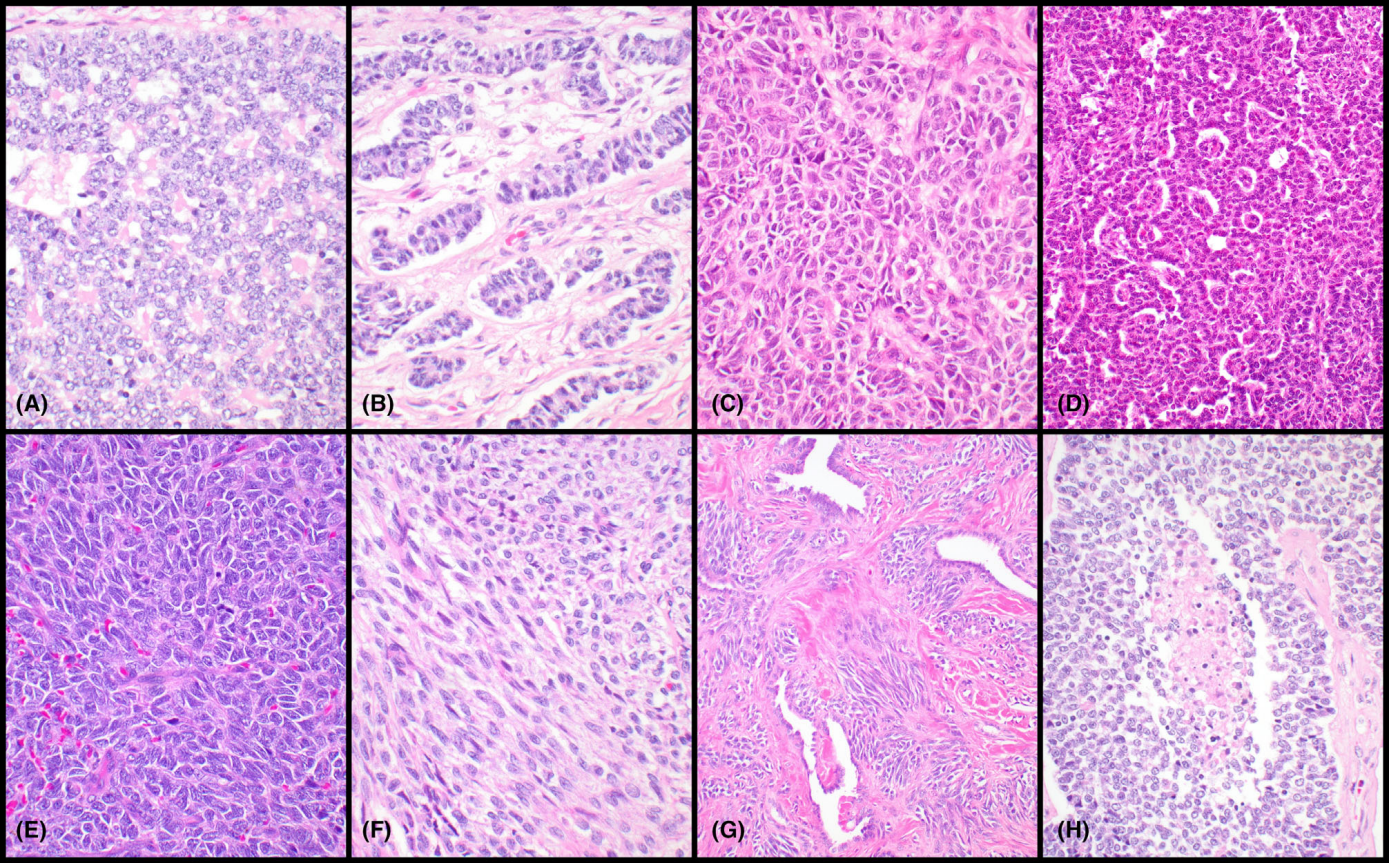
Adult granulosa cell tumours of the testis, common growth patterns. These tumours showed multiple growth patterns including microfollicles (**A**), cords/solid tubules (**B**), nests (**C**), microfollicles containing aggregates of tumour cells/glomeruloid structures (**D**), solid sheets (**E**) and fascicles of spindle cells (**F**). Note the presence of nuclear grooves in (**E**). The tumour shown in (**G**) entrapped areas of the rete testis and the one shown in (**H**) exhibited focal necrosis. The areas shown in (**A**, **B, H**) correspond to a case with a gain‐of‐function *CTNNB1* mutation.

**Figure 2:**
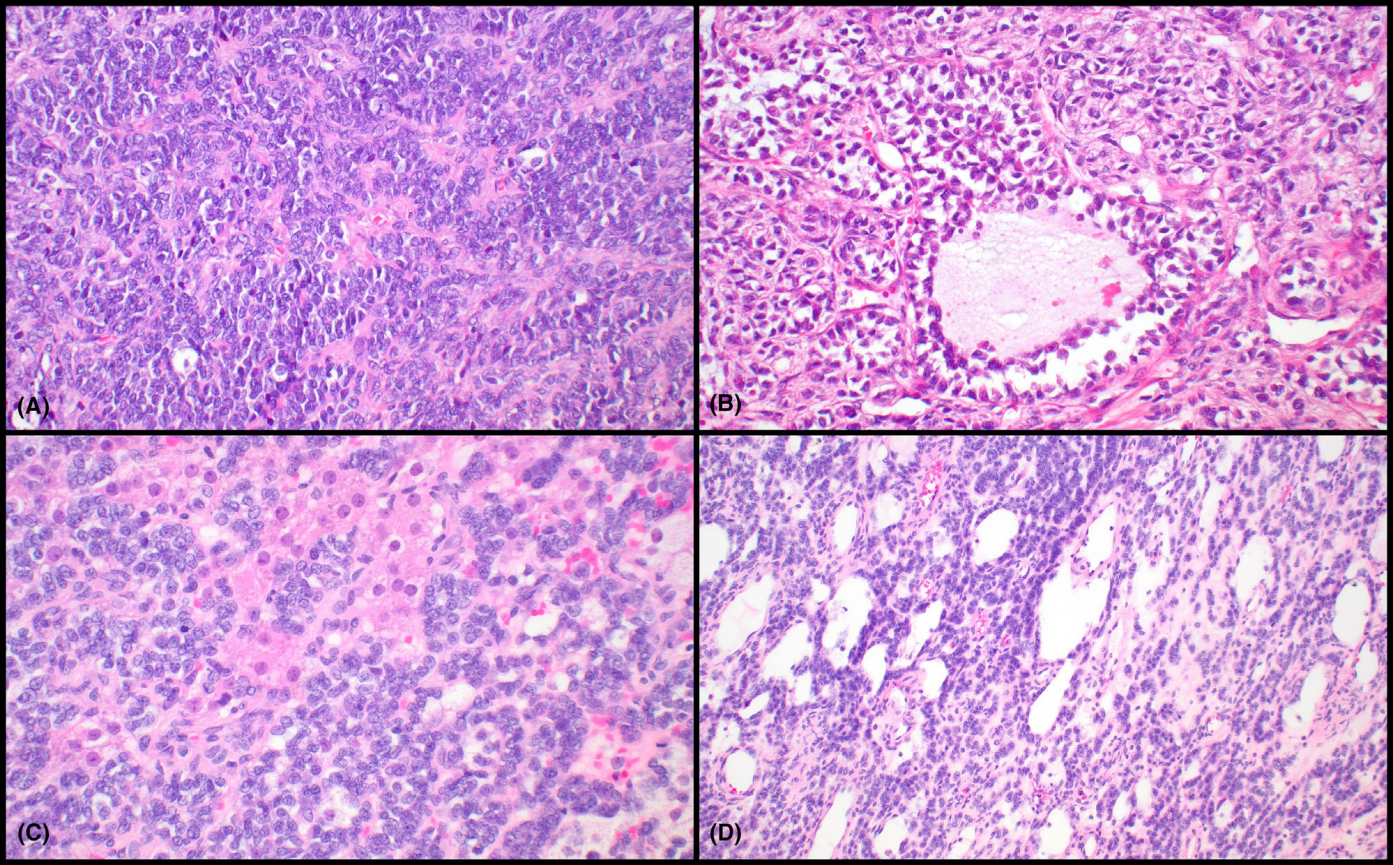
Adult granulosa cell tumours of the testis, unusual histopathologic findings (compared to prototypical ovarian counterparts). One tumour in the series showed areas with adult granulosa cell tumour morphology (**A**) and foci with juvenile granulosa cell tumour‐like morphology composed of follicles/cysts lined by small ovoid cells (**B**). Of note, this tumour had a cystic component (not shown). Two tumours with *CTNNB1* mutations showed foci with Leydig cell phenotype (**C**) and tubule‐like structures (**D**; the other *CTNNB1*‐mutant tumour is shown in Figure 1).

Twenty testicular AGCTs from 20 individual patients were included in this study. The patients showed a wide age range (22–71 years) with a median of 43.5 years (Table 1). Information about mode of presentation was only available for a subset of patients, including two with hydrocele, one with microscopic hematuria (i.e., incidental finding), one with a longstanding testicular mass and one with gynecomastia and mastodynia (Table 1). The median tumour size, available from the digital records only in 11 patients, was 30 mm (range: 8–95 mm). In 10/11 patients with available clinical history, a testicular mass was detected by physical and/or ultrasound examination. Three cases showed ‘worrisome’ histological features such as entrapment of the rete testis or necrosis (Figure 1). One tumour was clinically malignant (i.e., the patient developed metastases). Four additional patients with available follow‐up did not experience local or distant recurrences. The histologic appearance of these tumours spanned a relatively wide spectrum, including some with predominant spindle cell morphology. Micrographs of representative cases are included in Figures 1 and 2. Most neoplasms contained populations of small to intermediate‐sized ovoid cells with a limited to moderate amount of light eosinophilic, amphophilic or clear cytoplasm. Growth patterns included solid sheets, nests, trabeculae, cords, fascicles and microfollicles (Figures 1 and 2). Nuclei were irregular, with open chromatin, small nucleoli and frequent intranuclear grooves. One neoplasm (*CTNNB1* wild‐type) showed cystic areas and a combination of areas with AGCT and juvenile granulosa cell tumour‐like morphology (Figure 2). Re‐review of 3 *CTNNB1*‐mutant cases (i.e., identified after sequencing) showed the presence of tubule‐like structures in 2 of them (Figures 1 and 2), whereas the third one exhibited predominantly spindle cell morphology. Both *CTNNB1*‐mutant tumours with tubule‐like structures showed scattered foci with Leydig cell phenotype (Figure 2), and one also contained areas with microfollicular architecture. Immunohistochemistry performed as part of the original diagnostic workup was available for 16 cases (Table 1). All tested cases were positive for SF‐1 (7/7), WT‐1 (3/3) and FOXL2 (2/2), and most also expressed inhibin (13/14) and calretinin (9/11). Expression of smooth muscle actin (4/8) and keratins (7/12) was seen in significant subsets. SALL4 (0/4) and OCT3/4 (0/7) were negative in all cases in which they were performed (Table 1).

### NGS results and immunohistochemistry for β‐catenin

**Figure 3:**
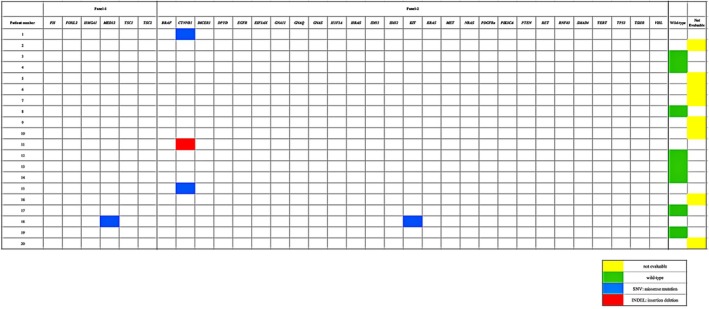
Genomic findings in adult granulosa cell tumours of the testis.

**Figure 4:**
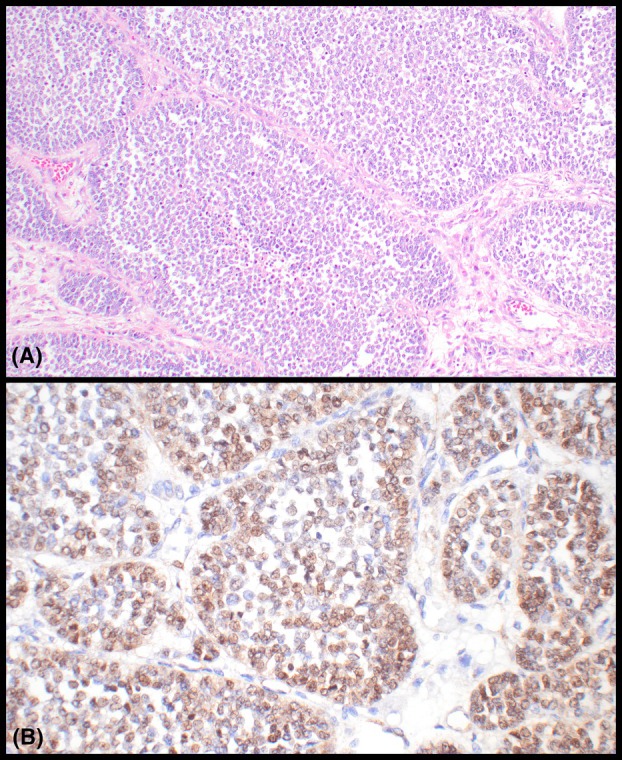
Adult granulosa cell tumours of the testis with *CTNNB1* mutation. This tumor showed predominantly solid nests of tumor cells (**A**; additional growth patterns present in this neoplasm are shown in Figure 1A,B,H). Nuclear β‐catenin expression was seen in ~70% of the tumour cells (**B**).

All tumours were sequenced *de novo*, including one that was previously assessed with fluorescence *in situ* hybridisation (FISH) and one that was previously tested for *FOXL2* mutations with Sanger sequencing in two prior studies.^14^, ^15^ Eight tumours (8/20; 40%) failed sequencing with both panels due to poor‐quality DNA and/or sub‐threshold quality assurance (QA) metrics.^10^, ^19^ None of the 12 tumours analysed successfully harboured *FOXL2* mutations. Eight tumours (8/12, 66.7%) showed a wild‐type (WT) status for all genes assessed with the panels. Among the remaining tumours, 3 harboured pathogenic or likely pathogenic *CTNNB1* alterations. The specific variants identified were *CTNNB1* c.110C>T (p.Ser37Phe, *case #1*), *CTNNB1* c.965_1012del (p.Gln322_Trp338delinsArg, *case #11*) and *CTNNB1* c.100G>A (p.Gly34Arg, *case #15*) (Table 2 and Figure 3). Specifically, the *CTNNB1* p.Ser37Phe and *CTNNB1* p.Gly34Arg mutations involve a hotspot region of exon 3, representing GOF events.^21^, ^22^, ^23^ The *CTNNB1* p.Gln322_Trp338delinsArg variant (exon 7), which affects the armadillo repeat domain (essential for proper protein folding), is likely pathogenic, although its biological significance requires further assessment in functional studies.^23^, ^24^, ^25^ Additional FFPE tissue was available for immunohistochemistry only in 1 of 3 *CTNNB1*‐altered tumours (*case #1*, Table 2), which showed β‐catenin expression in ~70% of tumor cell nuclei (Figure 4). Three different tumours (*cases #13*, *14* and *17*, Table 2) interrogated with β‐catenin immunohistochemistry as part of the original diagnostic workup showed absence of nuclear expression of the marker. Excluding the *CTNNB1*‐altered tumours described above, only one additional AGCT (*case #18*) harboured genomic variants: *MED12* c.3352G>A (p.Asp1118Asn) and *KIT* c.1694G>T (p.Gly565Val). Of note, the *KIT* variant has been variably annotated as ‘benign’ (https://varsome.com/) and ‘variant of uncertain significance' (VUS; https://www.ncbi.nlm.nih.gov/clinvar and https://franklin.genoox.com/). The *MED12* variant has been classified as VUS by Varsome and Franklin, while there are no prior entries in ClinVar. Overall, we speculate that these variants are most likely not relevant for oncogenesis in AGCT.

**Table 2:** NGS results and β‐catenin immunohistochemistry

Patient number	NGS results (p.)	NGS results (c.)	Exon	VAF	ACMG significance	Variant type	Immunohistochemistry for β‐catenin
1	*CTNNB1* (*p.Ser37Phe*)	c.110C>T	3	42%	Pathogenic	SNV	+/diffuse (70%)°
2	NE						NP^a^
3	WT						NP^a^
4	WT						NP^a^
5	NE						NP^a^
6	NE						NP^a^
7	NE						NP^a^
8	WT						NP^a^
9	NE						NP^a^
10	NE						NP^a^
11	*CTNNB1* (*p.Gln322_Trp338delinsArg*)	c.965_1012del	7	54%	VUS	INDEL	NP^a^
12	WT						NP^a^
13	WT						‐^b^
14	WT						‐^b^
15	*CTNNB1* (*p.Gly34Arg*)	c.100G>A	3	42%	Likely Pathogenic	SNV	NP^a^
16	NE						NP^a^
17	WT						‐^b^
18	*MED12* (*p.Asp1118Asn*)	c.3352G>A	23	13%	VUS	SNV	NP^a^
	*KIT* (*p.Gly565Val*)	c.1694G>T	11	52%	Benign/VUS	SNV	
19	WT						NP^a^
20	NE						NP^a^

Note: β‐Catenin expression was scored as previously described.^20^

Abbreviations: ACMG, American College of Medical Genetics and Genomics; INDEL, insertion/deletion; NE, not evaluable; NGS, next‐generation sequencing; NP, not performed; SNV, single‐nucleotide variant; VAF, variant allele frequency; VUS, variant of unknown significance; WT, wild‐type status.

^a^ Immunohistochemistry for β‐catenin was not performed because no residual material was available after NGS.

^b^ Immunohistochemistry for β‐catenin was performed as part of the original diagnostic workup.

### Literature review of the mutational landscape of testicular AGCTs

A literature review of PubMed‐indexed papers on testicular AGCT with molecular analysis published in English yielded only 6 studies.^10^, ^11^, ^12^, ^13^, ^14^, ^15^ These included testicular AGCTs analysed with single‐gene assays (*FOXL2*), massively parallel DNA sequencing, and fluorescence in‐situ hybridization (to assess numerical alterations of chromosomes 3, 6, 7, 9 and 11).^10^, ^11^, ^12^, ^13^, ^14^, ^15^ In total, 42 testicular AGCTs (including the ones analysed in the current study) have been assessed with Sanger sequencing and/or massively parallel DNA sequencing, of which 30 have yielded interpretable results.^10^, ^11^, ^12^, ^13^, ^14^, ^15^ *FOXL2* p.Cys134Trp has been identified in 2/29 (~7%) bona fide testicular AGCTs (one *FOXL2*‐mutant AGCT published by *Lima et al*. is excluded because it occurred in an intra‐abdominal gonad of a 46 XX patient with a male phenotype).^10^, ^11^, ^12^, ^13^, ^14^, ^15^ FISH and NGS studies have shown that testicular AGCTs with pure or predominant spindle‐cell components show a pattern of chromosomal gains similar to those seen in other SCST types with overlapping morphology (Table 3).^10^, ^14^

**Table 3:** Mutational landscape of testicular AGCTs (our case series and review of the literature)

Author and study	Number of testicular AGCTs tested (and with evaluable molecular results)	Adopted molecular test	Results
Schubert *et al*.^11^	1 (1)	PCR and pyrosequencing for *FOXL2* c.7558C > T (p.C134W) mutation	No *FOXL2* mutations detected (0/1)
Lima *et al*.^12^	5 (5); 4 (4) excluding the case occurring in the intra‐abdominal ovaries of a phenotypically male patient	PCR and direct sequencing of *FOXL2*	2/5 (40%) cases with *FOXL2* c.7558C > T (p.C134W) mutations^a^; however, 1 neoplasm was not a bona fide testicular tumor (see results section)
Hes^13^	3 (2)	PCR and direct sequencing of *FOXL2*	No *FOXL2* mutations detected (0/2)
Siegmund^10^	13 (10)	NGS (DNA panel, 447 genes)	*BRCA2*, *FOXL2* c.7558C > T (p.C134W) and *ATR*, *TP53*, *ATM*, *NRAS*, *CBFA2T3*, *ATR*, *ARID1B* mutations/co‐mutations each in 1/10 (10%) cases^b^ Recurrent copy number alterations in 9/10 (90%) cases, including: 22q arm‐level loss (7/10, 70%), 16q loss (3/10, 30%), single copy loss of chromosome 10 (3/10, 30%) and whole chromosome gain of chromosomes 3 (4/10, 40%), 7 (4/10, 40%), 9 (3/10, 30%) and 12 (5/10, 50%) No structural alterations/cancer‐relevant gene amplifications
Acosta^14^, ^c^	3 (3)^d^, ^e^	Combination of commercially available FISH probes (3q11.2, 6p24.3, 6q11.1, 6q23, 7q11.21‐q11.22, 9p21.3, 11q13.3, 17p11.2)	Gains of chromosome 3 in 1/3 (33.3%) cases, gains of chromosome 6 in 2/3 (66.6%) cases, gains of chromosome 7 in 2/3 (66.6%) cases, gains of chromosomes 9 in 1/3 (33.3%) cases and gains of chromosome 17 in 0/1 (0%) case^g^
Mesquita^15^, ^f^	1 (1)^f^	Sanger sequencing of *FOXL2*	No *FOXL2* mutations detected (0/1)
Present study	20 (12)	Two different NGS panels encompassing a total of 34 genes (*see Materials and methods*)	*CTNNB1* mutations in 3/12 (25%) cases; one case (8.3%) with co‐mutations of *MED12* and *KIT*
			No *FOXL2* mutations detected (0/12)

Note: Five previous studies and a total of 42 testicular AGCTs were included in this review (*see Materials and methods* and *Results—Literature review of mutational landscape of testicular AGCTs*).^10^, ^11^, ^12^, ^13^, ^14^

Abbreviations: AGCTs, adult granulosa cell tumours; FISH, fluorescence in situ hybridisation; PCR, polymerase chain reaction; VAF, variant allele frequency.

^a^ One *FOXL2*‐mutated case occurred in the intra‐abdominal ovaries of a phenotypically male patient.

^b^ One case with failed sequencing quality assurance metrics (average number of reads <50) harboured a pathogenic *BRCA2* mutation p.R2520* (coverage: 73 reads). The VAF of this variant was suggestive of a germline origin.

^c^ These cases were ‘spindle‐cell predominant’ (spindle cells >50% of the tumour volume).

^d^ Two cases (8 and 9 in this study) had undergone genomic analysis as part of the study by *Sigmund S et al*.^10^

^e^ One additional case of this study has undergone genomic analysis as part of the present study.

^f^ This case has undergone genomic analysis as part of the present study.

^g^ FISH for gains of chromosome 17 failed in 2/3 cases and was negative in the remaining case.

## Discussion

Testicular AGCTs are rare tumours that affect patients within a wide age range.^1^, ^2^, ^3^ Prior studies have suggested that they are molecularly and pathologically heterogeneous, with a low frequency of *FOXL2* p.Cys134Trp mutations when compared to ovarian counterparts.^7^, ^8^, ^9^, ^10^, ^11^, ^12^, ^13^, ^14^, ^15^ In practice, testicular AGCT represents largely a diagnosis of exclusion. Hence, AGCT has been probably used as a default diagnostic category for tumours that could not be classified into other specific subtypes (e.g., Leydig cell tumour, Sertoli cell tumour), resulting in significant heterogeneity. Consistently, a recent study showed that a diagnosis of AGCT is not reproducible even among genitourinary pathologists.^26^, ^27^ From a developmental perspective, testicular AGCT should represent an exceptionally rare event, since normal testes lack granulosa cells.^16^, ^17^ In Sertoli cells of murine testes, a switch to a granulosa cell‐like phenotype can be induced by forced overexpression of β‐catenin.^17^ Also, mice with constitutively active *TGFBR1* develop granulosa cell tumour‐like neoplasms.^28^ In practice, true heterologous SCSTs are rare, and their oncogenic mechanisms remain poorly understood. For instance, most ovarian tumours with a Sertoli cell‐like component are mixed neoplasms, such as Sertoli‐Leydig cell tumour and gynandroblastoma, including subsets associated with the *DICER1* tumour predisposition syndrome.^29^, ^30^ By contrast, neoplasms interpreted as pure ovarian Sertoli cell tumours are exceedingly rare and may show somatic *DICER1* mutation and/or occur in patients with Peutz‐Jeghers syndrome, associations that have not been described in tetsicular Sertoli cell tumor not otherwise specified.^30^, ^31^, ^32^ The occurence of some of these neoplasms in the ovaries of patients with Peutz‐Jeghers syndrome suggests that they are driven by *STK11* (rather than *CTNNB1*) alterations and may represent part of the spectrum of sex cord tumour with annular tubules.^30^, ^31^, ^32^ Similarly, we believe that most testicular neoplasms that are currently classified as AGCT do not represent true analogs of ovarian AGCTs.^10^ The results of the present study support this conclusion by showing that *FOXL2* p.Cys134Trp, the ubiquitous driver of ovarian AGCT, is typically not found in testicular AGCT. Three tumours in this study harboured pathogenic *CTNNB1* variants, which are not a feature of ovarian AGCT.^33^ Two of the *CTNNB1*‐mutant testicular AGCTs showed focal tubular morphology, but were not readily recognizable as Sertoli cell tumor, not otherwise specified. Immunohistochemistry performed on one of these neoplasms showed expression of nuclear β‐catenin (~70%) of a lesser extent than expected in Sertoli cell tumour, not otherwise specified (typically close to 100%).^34^ Notably, *CTNNB1* alterations are not specific for the latter tumor type, being found at variable frequencies in other testicular SCSTs, including Leydig cell tumor.^34^, ^35^, ^36^ Interestingly, *Ray LJ et al* have recently described a series of 80 ovarian AGCTs containing scattered tubules that show a significantly lower frequency of *FOXL2* p.Cys134Trp than typical AGCTs (2 of 11 cases that were analysed).^37^ However, none of them harboured *CTNNB1* alterations, suggesting that the two neoplasms mentioned above are unlikely to represent testicular counterparts of these ovarian neoplasms.^37^ This study has significant limitations that need to be briefly addressed. First, relatively small sequencing panels were performed. We chose this approach because our analysis was focused on assessing the status of *FOXL2* and other genes relevant for SCST (such as *FH*, *DICER1* and *CTNNB1*; all covered by the panel). Second, the rate of unsuccessful sequencing was higher than in prior reported series [8/20 (40%) tumours failed with both panels], which is unavoidable, given the average age of the material (Data S1).^10^ Third, additional tissue was not available for performing confirmatory β‐catenin immunohistochemistry in 2 cases with *CTNNB1* alterations detected by NGS. Finally, this is a retrospective study including a small number of patients. Notwithstanding the above drawbacks, this study yielded informative results. Specifically, it confirmed that testicular SCSTs classified as AGCT are significantly different from ovarian counterparts in that they largely lack the ubiquitous molecular driver *FOXL2* p.Cys134Trp. These findings reinforce the hypothesis that tumours historically diagnosed as testicular AGCT comprise a heterogeneous group, likely because AGCT has been used as the 'default' diagnosis of exclusion for SCSTs of the testis. Importantly, our results support the current WHO recommendation that testicular AGCT should be a rare diagnosis, ideally restricted to neoplasms analogous to prototypical ovarian counterparts.^26^, ^27^

## Author contributions

All the authors performed the study concept and design and wrote the manuscript; Costantino Ricci, Dario de Biase, Thais Maloberti, Antonio De Leo and Andres Martin Acosta contributed to the histologic revision of the cases and provided analysis and interpretation of data; Andres Martin Acosta provided clinical data and clinical analysis of the cases; Costantino Ricci, Dario de Biase, Andres Martin Acosta, Michelangelo Fiorentino and Giovanni Tallini performed the development of methodology and statistical analysis, and provided analysis and interpretation of data; all the authors performed the revision of the manuscript.

## Funding information

This paper did not receive any specific grant from funding agencies in the public, commercial or not‐for‐profit sectors.

## Conflicts of interest

The authors have no conflicts of interest or funding to disclose.

## Supporting information


**Data S1.** NGS sequencing success according to year of sample collection and the type of formalin used.

## Data Availability

The data that support the findings of this study are available from the corresponding author upon reasonable request.
