# Supplementary material for: Adult granulosa cell tumours of the testis analogous to ovarian counterparts are exceptionally rare: analysis of a multicentric series and review of the literature
Source: Histopathology. 2025 Nov 20;88(4):831–42. doi: 10.1111/his.70048 (PMC12891927; doi:10.1111/his.70048)
Supplement: Supplementary file 1 — Data S1. NGS sequencing success according to year of sample collection and the type of formalin used. [file HIS-88-831-s001.docx]

**Supplementary Material 1.
Correlation of NGS sequencing success with the years in which the tissues were collected and the type of formalin used.**

| Patient number | NGS sequencing success | Years in which the tissue was collected | Type of formalin adopted |
| --- | --- | --- | --- |
| 1 | Yes [*CTNNB1* (*p.Ser37Phe*)] | 2020 | 10% neutral buffered formalin |
| 2 | No (NE) | 2023 | 10% neutral buffered formalin |
| 3 | Yes (WT) | NA | NA |
| 4 | Yes (WT) | 2019 | 10% neutral buffered formalin |
| 5 | No (NE) | 2014 | NA |
| 6 | No (NE) | 2010 | NA |
| 7 | No (NE) | 2009 | NA |
| 8 | Yes (WT) | 2003 | NA |
| 9 | No (NE) | 2002 | NA |
| 10 | No (NE) | 2006 | NA |
| 11 | Yes [*CTNNB1* (*p.Gln322_Trp338delinsArg*)] | 2013 | NA |
| 12 | Yes (WT) | 2017 | NA |
| 13 | Yes (WT) | NA | NA |
| 14 | Yes (WT) | NA | NA |
| 15 | Yes [*CTNNB1* (*p.Gly34Arg*)] | 2020 | 10% neutral/phosphate buffered formalin |
| 16 | No (NE) | 2012 | NA |
| 17 | Yes (WT) | 2024 | 10% neutral buffered formalin |
| 18 | Yes [*MED12* (*p.Asp1118Asn*) and *KIT* (*p.Gly565Val*)] | NA | NA |
| 19 | Yes (WT) | 2017 | 10% neutral buffered formalin |
| 20 | No (NE) | 2013 | NA |

next-generation sequencing (NGS); not evaluable (NE); wild-type status (WT); not available (NA);
